# 3D Printing, Intellectual Property Rights and Medical Emergencies: In Search of New Flexibilities

**DOI:** 10.1007/s40319-022-01235-1

**Published:** 2022-09-01

**Authors:** Rosa Maria Ballardini, Marc Mimler, Timo Minssen, Mika Salmi

**Affiliations:** 1 Professor, University of Lapland, Faculty of Law, Rovaniemi, Finland; 2 Senior Lecturer in Law, City Law School, City University London, London, UK; 3 Professor, Center for Advanced Studies in Biomedical Innovation Law (CeBIL), University of Copenhagen, Faculty of Law, Copenhagen, Denmark; 4 Assistant Professor, Department of Mechanical Engineering, Aalto University, Espoo, Finland

**Keywords:** Pandemics, Supply chain regulation, 3D printing, Intellectual property rights, IP exceptions

## Abstract

The COVID-19 pandemic has exponentially accelerated the use of 3D printing (3DP) technologies in healthcare. Surprisingly, though, we have seen hardly any public intellectual property right (IPR) disputes concerning the 3D-printed medical equipment produced to cope with this crisis. Yet it can be assumed that a great variety of IPRs could potentially have been enforced against the use of various items of equipment printed out without express consent from IP holders. Many reasons might have motivated IP owners not to enforce their rights during the pandemic, such as the fear of acquiring a bad reputation during a declared situation of national emergency. There is no internationally recognised general exception to IPR enforcement for health emergencies, while several − sometimes ineffective − tools, like compulsory licensing, voluntary licensing arrangements and potential TRIPS waivers, have been considered or used to facilitate access to and the distribution of innovations in critical situations. During the COVID-19 emergency, this has meant that the 3DP community has been operating in a state of relative uncertainty including with regard to the risks of IP infringement. This study contextualises these issues for pandemic-relevant 3DP. Building upon experience gathered during the COVID-19 pandemic, we look to the future to see what novel mechanisms within the IPR system could provide the additional flexibility required for dealing more smoothly, with the help and support of digital technologies, with situations such as global health emergencies.

## Introduction

Over the past few years, 3D printing (3DP), a process of converting data from digital files into 3D models using a machine that prints layer upon layer of a selected material, has become increasingly important in healthcare. These developments have been driven by some of the main advantages of this technology, such as the fact that it enhances spare part availability and on-demand production, as the digital element of 3DP reduces both the costs and the difficulties associated with physical storage and the shipping of tangible products and spares.^1^^,^^2^^,^^3^ Traditional manufacturing processes work by removing material from a billet and/or require special tooling for making parts. Not having to meet those requirements makes 3DP faster at bringing products to market and entails fewer resources and less waste. In addition, starting the manufacturing process via 3D printing is much easier than with any other manufacturing method. 3DP is also already very widespread, even in homes. This challenges current intellectual property rights (IPRs), as it makes manufacturing easier and more widely available than ever. It is no coincidence that the COVID-19 pandemic has exponentially accelerated the use of 3DP in healthcare, as many schools, universities, organisations and individuals have combined forces to print the equipment needed to protect key workers as well as private individuals. The agility of 3DP for delivering and producing products gained momentum during the first waves of the pandemic in particular, with the 3D printing community stepping up by offering their services to ease pressure on governments and broken supply chains. Alongside COVID-19, the story of Dr Tarek Loubani – a Canadian-Palestinian doctor who used 3DP technology to create a stethoscope to overcome the shortage of medical devices in Gaza – is one of many famous examples that show how 3D printing technology can overcome shortages of essential goods where supply chains have broken down.^4^

Yet all this might not come without legal controversy, notably in relation to IPRs in the key technologies and innovations involved. For instance, disputes might arise when the ownership of inventions and creations that are 3D printed is seen to conflict with claims to IPRs related to those innovations. Surprisingly, with COVID-19, we have (with a few exceptions) seen hardly any public IPR disputes over 3D-printed medical equipment produced to cope with the health crisis.^5^ Yet it is difficult to see how IPRs were not relevant for any of the equipment printed out (without express consent) by the 3DP community. The issues are well showcased by the story of the alleged threat of patent infringement litigation by a medical device manufacturer against Italian engineers who reverse-engineered and produced patented valves with 3DP technology for use in a hospital in North Italy at the beginning of the COVID-19 pandemic. While the manufacturer denies having made such a threat, this still shows that IPR issues need to be considered and are not merely hypothetical.^6^ It appears that, in other cases, reasons such as the fear of acquiring a bad reputation during a declared situation of national emergency, or corporate social responsibility policies, have led IP owners not to enforce their rights during the pandemic. Most aspects of the COVID-19 response involving 3DP, including medical equipment, tracking systems, and software, as well as vaccines, diagnostics, and therapeutics, were subject to some form of exclusive right in respect of the IP involved, which was at times reproduced without explicit consent.

As of today, in fact, there is no internationally recognised general exception that allows IPR enforcement to be waived in the event of health emergencies or pandemics. At the same time, however, while tools such as compulsory licensing, combined with various types of voluntary licensing arrangements, are often referred to as facilitating access to and the distribution of innovations in critical situations like the one in question, most suffer from multiple shortcomings: for example, from burdensome, slow and time-consuming administrative processes (e.g. in the case of compulsory licensing) or from fragmentation and uncertainty (e.g. in the case of most voluntary licensing arrangements). This then raises the interesting legal and policy question whether and to what extent IPR owners *should* be entitled to enforce their rights with regard to critical products or processes in times of medical emergency such as global pandemics. For instance, in the specific context of 3DP and medical products related to COVID-19, the fact that there was no general exception for health emergencies or pandemics has meant that the 3DP community has had to take on the remarkable risk of being sued for IP infringements for printing without express permission. Moreover, one could question whether – even if there were a general IPR exception or some degree of flexibility (or a similar solution) for pandemics or global crises – more players in the digital manufacturing supply chain would have been willing to jump in, thus further increasing the supply of critical products and spares.

In view of scientific predictions that quite clearly link the worrisome speed of environmental degradation to a possible increase in global health pandemics, it is not so sci-fi to think that the next global health crisis is not very far away: what will happen then? Will IP owners still stay calm and quiet as they did for COVID-19? What if the next pandemic comes as soon as COVID-19 is over, for instance: is it even realistic to think that such a *laissez-faire* attitude by IP owners will continue in the same way? And if not, but, instead, IP owners aggressively enforce their rights with regard to the reproduction of essential medical equipment, such as that produced via technologies like 3DP, how will this affect the availability of such equipment as well as the potential to save lives?

This article contextualises these key issues in relation to the role of digital manufacturing technologies like 3DP in the fight against global health emergencies and crises. We take the COVID-19 pandemic and its implications as a case in point where relevant data is already available. At the same time, we look to the future to see what adjustments to the IPR system might be possible or even desirable in order to make best use of these decentralised digital technologies that enable local production, increase manufacturing capacity and provide considerable advantages for supporting production in the event of broken supply chains. We begin by presenting an overview of how 3DP technologies have helped in the fight against the COVID-19 pandemic. We then explore the role that the IP tools traditionally used in health emergencies, such as compulsory licensing and certain forms of voluntary licensing, have played during COVID-19 in the context of 3DP. This analysis sheds light on the main deficiencies in our current IP system in terms of promoting the use and development of digital manufacturing technologies during periods of emergency. We then build upon this analysis to develop novel mechanisms that could be further explored and tested within the IP system in order to give the system the flexibility it requires to deal more smoothly, with the help and support of digital technologies, with global health emergencies and other similar situations.

## Medical 3DP and Pandemics: Past, Present and Future

3DP can be used to produce various medical devices, from patient-specific medical models and implants to instruments and parts for these same devices.^7^ The 3DP process always requires a 3D model of the object to be manufactured. In medical applications, personal geometry is often based on medical imaging or 3D scanning, but 3D modelling can also be used on its own.^8^

At the beginning of the COVID-19 pandemic, there was a shortage of various medical supplies because of both high demand and supply-chain breakdowns caused by national and global restrictions and lockdowns.^9^ All this forced nations to look for alternative, local, ways to manufacture certain supplies such as personal protective equipment, ventilators and consumables used in COVID-19 testing. Here, the advantages of 3DP stepped in: digitalisation helped narrow and shorten the supply chain, and local manufacturing enabled manufacturers to reduce waste in time and materials, as well as optimise costs. This reduced dependency on critical supplies from other countries and increased industrial resilience, as also noted by the European Commission.^10^

As a consequence, the development curve of medical 3DP technologies and applications during COVID-19 has been remarkable, with many solutions being made available as open-source options. At first, development focused on the 3DP of simple holders for face shields, door openers and other, very low-risk, simple parts.^11^ Then studies demonstrated how other personal protective equipment, such as face masks, or spares therefor, could be 3D printed. When COVID-19 diagnosis was ramped up there was a shortage of nasopharyngeal swabs for collecting clinical test samples, and 3DP was able to manufacture those as well.^12^ In addition, 3DP can be used for making prototypes to speed up the development of products – including those related to the pandemic. At the time of writing, the use of 3DP for improving devices used for manufacturing vaccines,^13^ and for producing other forms of personalised medications in both hospitals and in home settings is being explored.^14^ Clearly, such novel applications would not only help to achieve sustainability goals^15^ but could become very useful in any future pandemic or other health crisis.

Returning to the present crisis, it is hard to estimate what proportion of medical supplies has been 3D printed during the COVID-19 pandemic. But, for example, tens of millions of swabs have been printed on the basis of the 3D model and the process developed by the University of South Florida.^16^ As many similar 3D printing solutions have been implemented, the total amount of 3D-printed swabs could actually amount to hundreds of millions. The volunteer organisation Open Source Medical Supplies estimated that they alone produced 25 million face shields, mostly by 3D-printing the frame.^17^ Based on open-source solutions, the amount of similar devices worldwide is estimated at 4,000,000 nasal swabs, 3,000,000 venturi valves, 700,000 face masks and 1,000,000 face shield holders, which could have been 3D printed in a day, without counting any alternative 3DP devices and materials.^18^

The combination of 3DP technology, an open-source way of thinking and a willingness to share has clearly shown its potential in the COVID-19 pandemic.^19^ Overall, 3DP has become one of the new key technologies in combating the COVID-19 crisis. That said, it is not entirely clear to what extent such uses would have infringed IPRs, though it can be assumed that many cases could have ended up in litigation if the IP proprietors had chosen to initiate infringement proceedings. Looking ahead, it therefore appears warranted to analyse the flexibilities in IP legislation with regard to new technologies and health emergencies, to ensure that the risk of IP infringement does not slow down or even prevent effective pandemic responses in extraordinary times.

## Health Emergencies and Flexibilities in European IP Law: the Case of COVID-19 and 3D Printing

### Compulsory Licensing

Compulsory licensing is one of the regulatory mechanisms typically mentioned as offering ways to circumvent the exclusive effects of IP rights. Ordinary licensing involves a contract between the right holder and the party that is seeking to use the subject matter covered by the IP right. The conclusion of a licensing agreement grants the licensee the right to use the invention without infringing it, as use is then authorised by the right holder. Under compulsory licensing, this authorisation is replaced by the decision of a competent authority or court. The mechanism of compulsory licensing is clearly regarded as a last resort, i.e. when licensing negotiations between the parties are futile or if other factors stipulate non-consensual solutions. As Gordon says: “Although intellectual property has provided mechanisms to facilitate consensual transfers, at times bargaining may be exceedingly expensive or it may be impractical to obtain enforcement against non-purchasers, or other market flaws might preclude achievement of desirable consensual exchanges”.^20^

Compulsory licensing has been a feature of international IP law for some time now. Most notably, it was introduced, in relation to patent law, in Art. 5.A(2) of the Paris Convention.^21^ The contemporary international framework in the form of the World Trade Organization’s (WTO’s) Agreement on Trade-Related Aspects of Intellectual Property Rights (TRIPS) emphasises the role of compulsory licensing in patent law (Art. 31^22^ and Art. 31^bis^), while remaining silent on the compulsory licensing of other IP rights.^23^ Article 31 TRIPS lays down the procedural and substantive conditions under which compulsory licensing of patents is possible, but sets only minimal standards,^24^ allowing states to devise more stringent criteria. The conditions laid down in Art. 31 TRIPS include prior attempts to obtain licences on a voluntary basis from the patent holder (which, however, may be waived by member states “in the case of a national emergency or other circumstances of extreme urgency or in cases of public non-commercial use”), determination of the scope of the compulsory licence, its non-exclusivity and non-assignability, and making it subject to adequate remuneration. These conditions stipulate a restrictive application and, as they follow Art. 30, mean that compulsory licensing should be applied only in exceptional circumstances, with a patentee’s unfettered exercise of its rights being the rule. This restrictive approach was softened following the Doha Declaration on TRIPS and Public Health of 2001 and subsequent events, which led to the introduction of Art. 31^bis^ of the Agreement.^25^

Some countries have issued special legislation regarding compulsory licensing in response to the COVID-19 pandemic. Israel, for instance, has granted a compulsory licence under Art. 104 of the Israeli Patent Act in order to import a generic version of AbbVie’s Kaletra from India. The provision does not foresee prior consultation with the patent owner, nor does it permit judicial review of the decision.^26^ Similarly, Germany amended its Act on the Prevention and Control of Infectious Diseases in Humans in early 2020 to address the challenges posed by the COVID-19 pandemic. Some of the amendments delegated special powers to the Federal Minister of Health in the event of an “epidemic situation of national significance” (Sec. 5 of the Act), including the power to use a patented invention pursuant to Art. 13 of the German Patent Act. In contrast with the compulsory licence granted under Art. 24 of the German Patent Act, Art. 13 does not grant the right to use a patented invention to a particular licensee but rather provides that that the patent holder cannot exercise their right by prohibiting acts covered by an order issued pursuant to Sec. 5(2), No. 5, of the Act on the Prevention and Control of Infectious Diseases in Humans. While such an order can be challenged by the administrative courts, it would not have suspensory effect.^27^

Applying compulsory licensing as a fall-back solution in medical emergencies has substantial drawbacks, which makes it futile as an option in the context under discussion. Firstly, the time frame for overcoming the procedural obstacles to the grant of a compulsory licence is not suitable for addressing a healthcare emergency. The fact that the amendments to Art. 31 TRIPS after the Doha Declaration only led to one successful application, when Rwanda notified the WTO’s TRIPS Council of its intention to obtain generic HIV drugs from the Canadian company Apotex, highlights its limited use in a heath emergency. In that case, the whole process was perceived as cumbersome,^28^ and the drugs destined for Rwanda took 15 months to arrive there. Thus, the advantage that 3D printing technologies provide would be lost in a web of procedures and possible litigation by right holders that may wish to oppose the grant of a compulsory licence. As seen, some compulsory licensing procedures can be conducted without notifying the patent holder, as indeed the TRIPS Agreement specifically allows. But this usually involves government institutions in some form and does not seem to allow private individuals or entities to act unilaterally.

Secondly, compulsory licensing does not cover know-how and, in particular, tacit knowledge relating to the production of IP-protected goods. These may be covered by trade secret protection and non-disclosure agreements (NDAs). This aspect is particularly relevant in the context of vaccines^29^ but may also apply to other more complex and sophisticated goods that can be replicated by 3DP technologies. “Reverse-engineering” such knowledge is burdensome and time consuming and would require much trial and error. Thirdly, compulsory licensing is generally available for patented technologies but not necessarily for other IP rights that may be relevant for reproducing medical technologies. For instance, the EU design framework currently does not provide for compulsory licensing, although this is not prohibited by the TRIPS Agreement.^30^ The same applies, with some very minor exceptions, to data and market exclusivity rights, which might be relevant for some medical devices, products and compounds.^31^ Fourthly, compulsory licensing of an IP right is tethered to the territoriality principle of IP rights.^32^ This may require applying for multiple compulsory licences in different jurisdictions that apply different procedures. All in all, compulsory licensing, often proffered as a possible remedy, is something of a “lame duck”.^33^

#### Interim Conclusion

While compulsory licensing is sometimes mentioned as providing access to protected technologies, its drawbacks severely dampen its role as a viable solution for health emergencies in the context under discussion. The advantage of the speed with which 3DP could be deployed to address shortages in medical equipment and other tools by leap-frogging supply chains would be cancelled out by the burdensome administrative hurdles that potential users need to overcome in order to commence production without the chilling effect of facing potential IP infringement claims by right holders. Furthermore, the facts that compulsory licensing is currently not available for all IP rights that could potentially be affected by 3DP, that know-how is not covered, and that there is a territoriality issue, as mentioned above, make compulsory licensing – as a sole solution – a rather clumsy and inept mechanism to deploy in medical emergencies.

### Voluntary Licensing: IP Pools and Pledges

Generally speaking, voluntary licensing is the naturally preferred mechanism for disseminating life-saving technologies. It is built on the main tenets of IP right protection: exclusivity and freedom of contract between the parties. It is also deemed to be the rule because, as previously noted, the TRIPS Agreement prescribes that compulsory licensing can usually only be pursued if voluntary licensing has failed.^34^ Looking specifically at the COVID-19 pandemic and the way that voluntary licensing has been used in relation to 3DP, two main tools emerge, namely IP pools and IP pledges.

#### IP Pools

IP pools – or joint licensing – are agreements between two or more parties to cross-license parts of their current or future IP portfolios relating to certain technologies to one another or to third parties.^35^ Typically, such licensing is via mutual coordination or via a third-party administrator. IP pools are especially appealing when dealing with complex and interoperable technologies, where multiple players can contribute their own complementary strengths. It is worth mentioning that, although IP pools were initially viewed with hostility by regulators due to their potential clash with competition law, the competition authorities now accept them, if with some reservations.

In the context of COVID-19, patent pools have developed in general – for example, the recently signed Medicines Patent Pool (MPP) agreement related to the manufacture of a diagnostic kit.^36^ In the specific context of COVID-19 and related 3DP applications, the “COVID 3D TRUST: Trusted repository for users and suppliers testing”^37^ created in the USA is a type of IP pool. The COVID 3D TRUST is hosted under the NIH 3D Print Exchange portal, which is a resource from the National Institute of Allergies and Infectious Diseases (NIAID), and is curated by the National Institute of Health (NIH) and NIAID, in collaboration with the US Food and Drug Administration, the Veterans Healthcare Administration, and America Makes. As stated on the website: “This collection is intended to support innovation and informed decision-making during the COVID-19 pandemic, while critical safety and medical equipment are unavailable through traditional supply chains”.^38^ The COVID 3D TRUST pool relies on the same principles and licences as the NIH 3D Print Exchange portal, in that it is an open repository for finding, sharing, and creating (certain types of) 3D-printable models, and uses a variety of licensing options, all of which are of open-source type.

What is particularly interesting in this example is that the designers are not only sharing their (possibly IP-protected) technologies and designs, but are also responsible for providing “thorough instructions to help others reproduce [them] accurately”.^39^ In other words, this arrangement might – at least partly – overcome one of the main criticisms of IP pools, namely that they are venues for sharing only selected IP-protected technologies, while know-how is kept outside of the pool. Forcing designers to share also some of their know-how might create a better venue for fostering technology transfer and building capacities. Another interesting point about this type of pool is that it is – again – not a typical “closed-doors” scenario between selected organisations that have concluded agreements with each other but rather an open venue for sharing, where anyone can contribute. Indeed, it relies on open-source-type licences, which enable this more agile and open model of operation. In a way, this type of arrangement is very representative of a large part of the 3D printing and manufacturing community, which relies heavily on the concepts of sharing and co-creating. At the same time, however, it is clear that these kinds of arrangement are an impediment to commercialisation when open-source licences like the GNU General Public License are used – which might indeed make most commercial organisations’ motivation for sharing questionable and open to challenge. In addition, monitoring whether or not the solutions offered in such a pool will actually work is highly complex and challenging. The ambitions of individuals also drives similar technical solutions with a slightly modified design, which might lead to too many similar options being offered, with the best ones being obscured behind too many possible alternatives. Finally, this system carries the risk that people with limited skills and experience could manufacture certain goods that might not even work as expected.

#### IP Pledges

Another type of voluntary licensing mechanism that has been used during COVID-19 in the 3DP context is what is known as the “IP pledge”. IP pledges are publicly announced interventions by IP owners to out-license active patents (or, in general, IPRs) for a certain period of time, either “free from or bound to certain conditions for a reasonable or no monetary compensation”.^40^ Depending on the conditions, pledges might not necessarily benefit only certain pre-determined groups of players that have made formal agreements amongst each other, but could also apply to the wider public unconditionally.^41^

One example relevant to the COVID-19 pandemic and 3DP, for instance, is the patent pledge announced by University of South Florida Health and Northwell Health for their 3D-printed nasal swab.^42^ This pledge concerns access to a protocol and links related to making the 3D-printed Nasal Swab, a novel invention developed by a USF researcher for emergency situations arising out of the COVID-19 public health emergency. This pledge states that “[t]his 3D print swab design protocol is patent protected. The University of South Florida grants the recipient of design files permission to 3D print and use the swabs for non-commercial purposes until April 15, 2021”. The files, pledge and documents are not available online any more but may be obtained on request.

Another interesting example on a larger scale is the Open COVID Pledge, an initiative originally launched by “an international group of researchers, scientists, academics and lawyers seeking to accelerate the rapid development and deployment of diagnostics, vaccines, therapeutics, medical equipment, and software solutions in this urgent public health crisis”. This initiative is led by the Program on Information Justice and Intellectual Property at American University Washington College of Law.^43^ Anyone can join the pledge, so in a way the Open COVID Pledge provides more of a venue for pledging. So far, many of the largest technology companies – including Amazon, Intel, Microsoft, Hewlett Packard Enterprise and Facebook – have signed the pledge. A wide range of licences may apply, as there is no required form of licence that pledging companies must agree to use. A set of three similar licences was created for the Open COVID Pledge, referred to as an “Open COVID License”. However, there is no standard licence that a licensee can assume controls the licensing. Each licence other than the three versions of the OCL may contain any number of custom terms. In fact, as of today, there are already several other possible licence terms that may apply, which makes it crucial that all licences be reviewed individually. These licences are intended to be effective as of 1 December 2019 and to last for one year after the WHO declares the COVID-19 Pandemic to have ended – but in any event not beyond 1 January 2023, unless otherwise extended by the Pledgor. For the 3DP side, for instance, the Open COVID Pledge features two examples so far: (1) NASA has pledged rights in relation to 3D-printed respirators, including relevant instructions, 3D models and initial test data; and (2) the New Jersey Institute of Technology has pledged a 3D-printable forceps swab for COVID-19 testing, designed to reduce infection and the risk of contamination, including relevant instructions and 3D models.

Indeed, voluntary pledges to make IPRs broadly available can overcome administrative and legal hurdles faced by more elaborate legal arrangements such as IP pools, and can achieve broader applicability and greater acceptance than tools such as governmental compulsory licensing.

Notwithstanding the possible advantages, however, IP pledges do also seem to suffer from some limitations. One main concern relates to the fact that conditions for pledging are dependent on the wishes of the IP owner (the pledgor), so in a way they are unilateral agreements. Such conditions vary considerably from one pledge to the other, creating fragmentation and uncertainty, and increasing complexity. For instance, with the Open IP Pledge initiative there is no obligation for participating organisations to license an entire patent or copyright portfolio, and each company that pledges is free to exclude any of its patents or copyright.^44^ All this makes it imperative to check the scope of the IP licensed by each pledgor. This might become a heavy burden when several licensing terms and conditions are in use. Another important downside of IP pledges is that they are temporary – as seen in the cases of both the 3D-printed Nasal Swab and the Open COVID Pledge. This might actually lead to a lock-in situation, where organisations that include temporarily pledged, IP-protected technologies in their own solutions might be forced to enter into licensing negotiations when the pledge expires. This is especially the case for COVID-19 pledges, which usually apply only if the IP is used in relation to COVID-19 (e.g. for developing COVID-19 diagnostic, prevention or treatment products). Thus, if the IP is included, for example, in other products that can be used for other viruses, there is a risk that such use will exceed the scope of the licence. It should also not be forgotten that pledges do not obviously include any of the protection normally obtained through commercially negotiated licences. Last but not least, IP pledges entail the same general problems as compulsory licensing or IP pools in terms of enabling the sharing of know-how or developing local infrastructure, because what is also true of pledges is that what is licensed out is, in most cases, only the IP, while both the know-how and training capacities are normally not shared. To sum up, even if IP pledges are a quick fix and do not involve heavy administrative tools, thus allowing different players to develop solutions around a common technological core, they might be too limited when we look at the larger multi-technology and multi-purpose innovation ecosystems often required in health emergency cases.

#### Interim Conclusion

All in all, while the advantages of voluntary licensing mechanisms are clear, there are also several pitfalls. In addition to those mentioned above, which are specific to each type of voluntary licensing tool, voluntary licensing includes more general downsides in the context of global pandemics, especially in relation to 3DP. These are worth mentioning. First, as IP is territorial, voluntary licensing tends to be limited to a particular territory.^45^ This does not help in the case of a pandemic, where multiple territories are hit equally.^46^ This gives the originator companies scope to negotiate differentiated terms for different territories. Second, another problem faced by voluntary licensing specifically in relation to 3DP is that it may constitute uncharted waters for originator companies. Many might have licensed their IP relevant to creating the technologies in question to other companies, which then manufacture the goods. They might also have assigned their relevant IP rights to other companies. However, companies that largely use conventional manufacturing processes are not yet aware of the possibilities provided by 3DP technology. This might then lead to uncertainty regarding the process of licensing their IP for manufacturing using 3DP technology, for example which rights need to be cleared and what royalty rate to ask for. Interestingly, such a development would arguably boost the market in spare parts. Originator companies could add digital inventories to their tangible inventories and add digital distribution of their IP to their business models.

## Fostering 3D Printing Developments and Uses in Future Health Emergencies and Crises Through IPR Flexibility

Our analysis confirms an old and well-known dilemma: IPRs in the health sector are both essential and potentially problematic. In particular during health emergencies, achieving a reasonable balance between protection and access to urgently needed innovations is a delicate task. The example of 3DP demonstrates how IPR can at times impede agile, flexible mobilisation of digital (manufacturing) technologies to provide the rapid and reliable solutions needed during emergency situations. Lengthy and highly political negotiations on possible solutions to the “IP problem”, like those surrounding the hotly debated “IP Waiver”,^47^ demonstrate once again that preparedness and proactivity in IP law need to be debated proactively – not reactively after a crisis has already emerged. Moreover, what also transpires from the above is that some of the tools and structures of the current IP system are in need of further development and refinement.

No doubt this is a complex problem with no single bullet-proof solution. Putting all eggs into one basket – as seems to be the hope of some proposed “solutions” such as the IP Waiver – is most likely not a functional or sustainable way to proceed and has seen much opposition from governments in developed countries. The Decision by the Ministerial Conference of 17 June 2022^48^ thus produced no more than a significantly watered-down version of the original proposal made by India and South Africa, which would have suspended the obligation to enforce certain provisions of the TRIPS Agreement in order to allow the prevention, containment and treatment of COVID-19.^49^ The compromise now is limited to the production of COVID-19 vaccines, which would not involve 3DP technologies. After all, existing IPR mechanisms, like those presented in Sec. 3, already have the potential to embed flexibility in the exclusive IP rights framework. This flexibility should not be underestimated or disregarded. The question is rather whether there is a need and sufficient leeway to create even more innovative options for increasing flexibility in IPR in order to be better prepared for the next health crisis. What could these new tools be in the specific context of 3DP or digital manufacturing technologies? Below we shed some light on some prominent suggestions that could be explored in this context.

### New Exceptions in IPR

#### Health Emergency Exceptions

One intuitive legislative tool to swiftly grant time-limited access to and use of protected technology in times of medical emergency would be an exception provision that permitted such uses in medical emergencies. Thus, the chilling effect on players that wish to reproduce or otherwise use protected features by way of 3DP technologies but fear potentially infringing IP could arguably be eliminated or drastically diminished in times of crisis. If the requirements for application of the exception provision were clearly laid down, players could rely on it in infringement proceedings brought by the right holder against any alleged infringement. In addition to creating legal certainty linked to a robust definition of what constituted a medical emergency or “public health emergency”, another benefit of such an exception would be that users would not need to wait for the right holder’s authorisation when reproducing protected features or await the outcome of administrative procedures as is required under compulsory licensing.

From a policy perspective, exceptions also have advantages over other legislative mechanisms, such as excluding certain subject matter from IP protection. Such exclusions – for instance, those available for methods for treatment of the human or animal body by surgery or therapy and diagnostic methods practised on the human or animal body^50^ or for discoveries, aesthetic creations or computer programs as such^51^ in patent law – provide that this subject matter may not be protected by an IP right.^52^ Consequently, the relevant features would remain in the public domain, unencumbered by exclusive rights and free for anyone to use and reproduce. However, this might negatively affect research and development in these areas as the incentive mechanism of IP would no longer apply^53^ and developers might be motivated to keep their developments secret.^54^ This would in turn make it more tedious to reproduce such features by 3DP.^55^ Exceptions nevertheless enjoy the advantage that they do not fully remove the incentive mechanism that the IP right seeks to confer on right holders.^56^ Another disadvantage of exclusions is their nature as an *ex ante* mechanism. Legislators must decide *ex ante* what subject matter ought to be excluded, and under what conditions, such as a health emergency. But how can legislators ascertain *ex ante* which technologies would be required to remain in the public domain for use in medical emergencies? Exclusions may also be required horizontally in relation not only to patents but probably also other IP rights. But even where an exclusion could be drafted more narrowly by, for instance, excluding medical products from patent protection, this would arguably be in violation of the TRIPS Agreement and the principle of non-discrimination in Art. 27(1) thereof.^57^

The attractive option of legislating a general exception provision, however, has a high hurdle to overcome. The so-called “three-step test”^58^ for all IP rights within the TRIPS Agreement poses a significant barrier to the introduction of such an exception by legislators in WTO member states.^59^ In order to be applicable to and workable in the health emergencies discussed here, such as the COVID pandemic, any exception provision would need to be broadly drafted and flexible enough to encompass a plethora of permitted uses of a plethora of possible technologies and devices.^60^ An exception provision for patented inventions could, for instance, be drafted so that “the rights conferred by a patent shall not extend to any of the following: (a) acts done to address a health care emergency”.^61^ However, this might seal the fate of such provisions since such a broad nature would arguably conflict with the first step of the three-step tests, which in each case mandates that an exception must be limited. In the *Canada v. EC* decision of 2000,^62^ the Panel held that the term “limited” had a narrow meaning.^63^ It qualified the term “exception”, suggesting a “limited derogation” from the patent right, i.e. “one which makes only a small diminution of the rights in question”.^64^ Applying this rationale to the example here of the health emergency exception, the provision suggested would most likely not comply with the reasoning of the Panel in the *Canada v. EC* decision. As the test fails if one requirement of one step is not met, the entire exception provision would fail.^65^ The option of drafting a tighter and narrower exception provision that would limit permitted uses to digital manufacturing by 3DP would arguably also fail at the first step. As such an exception would not provide for any quantitative limitation on how many products could be produced under the exception, it is most likely to meet the same fate as the Canadian stockpiling exception that came under scrutiny in the *Canada v. EC* decision. This approach, coupled with the way the test is interpreted, might prevent legislators from enacting any exception provisions compatible with TRIPS for the purposes we mention since, unless this interpretation is dropped, no account at all is taken of the real-life economic impact on the right holder^66^ and the relevant policy considerations^67^ behind the exceptions.^68^

#### Private Use

Another option could be to try to enhance IPR flexibilities by better promoting the role of an Open Source (OS) vision and related activities. For instance, as previously mentioned, OS communities played a key role in the use of 3DP technology during the COVID-19 pandemic. In particular, two types of player emerged from the OS community:

1. Those who produced essential equipment (e.g. face shields) based on someone else’s instructions and donated them to e.g. hospitals and health care organisations.
2. Those who developed new innovations relying on 3DP technology (e.g. 3D-printed ventilators) to help with the pandemic crisis and licensed them out via open source licences.

While the second type is, in principle, unproblematic, the first type is a matter for concern. The activities carried out by players of the first type raise several possible infringement concerns when the items or methods they use – or parts thereof – are protected by IPRs. As previously mentioned, this seems highly likely.

It is likely that players of the second type would not be caught by the private and non-commercial use exception as currently interpreted, because both conditions must be present for the exception to be applicable. In this regard, what could be considered is whether, for example, the private and non-commercial use exception could be interpreted in a way that could also apply to consumer-engaging technologies like 3DP during health emergencies where the conditions of both private and commercial use are not met. Mendis et al.,^69^ for instance, suggest that one way to encourage follow-on innovators to step in quickly in situations such as a pandemic could be to create a carve-out that would apply to private *or* non-commercial uses of technologies (like 3D printing), whenever needed during public (health) crises. This carve-out could become applicable upon declaration of an emergency by local or national authorities. This would allow copies of life-saving medical equipment to be made, as such manufacture would be exempt from liability for the duration of the crisis. To avoid abuse of the exception post-crisis, follow-on innovators would be forbidden from releasing the digital files containing the instructions for 3DP the required medical equipment, drugs, etc.^70^ Similar solutions would need to be considered if account had to be taken of other technologies. This would continue to encourage innovation, promote access to inventions, and maximise the use of 3DP and IPRs for the greater public good in times of health crises.

However, the clear challenge with this proposal is the lack of compensation for those who develop technology, especially when we consider inventions and patents. Moreover, the private and non-commercial use exception for 3DP in patent law has already raised many eyebrows. Historically, the private and non-commercial use exception in patent law has been quite uncontroversial, as use by private persons for non-commercial purposes (excluding research and experimental purposes) has been rare for both technological and economic reasons.^71^ Similarly, the activities of hobbyists and DIYers have traditionally attracted little attention from patent holders because their use of patented inventions is normally no more than one-off and, as such, does not fall within the market targeted by patent owners in the value chain. 3DP as a technology already challenges the rationale and acceptability of this exception, as it offers novel possibilities to reproduce inventions relying on the private and non-commercial use exception. With that in mind, it might be difficult to try to relax even further an already controversial exception for 3DP; thus, although this is an interesting solution, it might be quite difficult to gain acceptance for it.

### New Mechanisms to Foster Resilience During Pandemics

Consideration must be given to how to reduce the negative impact of pandemics in general, including effects on governments, companies and individuals. It might be important to include in such consideration other mechanisms that seek to optimise the mitigation of both monetary and health-related losses by fostering cooperation amongst stakeholders with a view to enhancing knowledge sharing and building up capacity. With intelligent cooperation, it could be possible to find win-win types of collaboration, instead of having IPRs, skills and capacity each held individually by different partners.

In this context, one option could be to take a similar approach to that taken, for example, by the mobile networks industry in relation to standard essential patents. Faced with the increasing problem of interoperability between various devices globally, companies opted against different basic network technologies between different manufacturers and instead for standardisation and the use of essential patents to create network compatibility through a mechanism of compensation by royalty payments under “fair, reasonable and non-discriminatory” (FRAND) licensing terms and conditions for the developer of a technology solution.^72^

As far as pandemics are concerned, the same approach and logic could, for example, operate as follows: If national governments predict shortages of specific IP-protected equipment that is declared “essential” for fighting an emergency (for example, ventilators or PPE in the case of COVID-19), organisations holding IP rights in respect of such products or methods would assign their rights to, for instance, specific collective management organisations under FRAND licensing conditions in order to enable manufacturing to be ramped up globally. There could be two options here: one could be to expand the scope of existing collective management organisations to include the management of licences for IPRs other than copyright, for example during exceptional circumstances, as when national health emergencies are declared; another option could be to create an ad-hoc collective management organisation for health emergencies. The former option might be more manageable as it would rely on already well-established institutions, thus reducing the costs of creating a new body. Regardless, it is clear that investment in further developing the structure of such a stream of licensing management and models of how it should work once a state of emergency has been declared should start immediately – not only after an emergency has arisen. Organisations working on innovations that are likely to be needed in health emergencies could also already start negotiating agreements with the collective management organisations, so that the licensing management mechanisms could be operative immediately once a state of emergency occurs.

This proposed model might be more complex to design and manage than currently existing similar systems in, for example, Europe. For instance, one of the complexities stems from the likely higher heterogeneity of the players from whom the rights to license the IPR-protected innovations would have to be acquired. While this would depend on the type of specific IP-protected equipment declared “essential” to fight the emergency at stake, it could, for example in the case of 3DP, encompass both equipment and material manufacturers, as well as potentially a variety of prosumers of the technology, such as DIYers, etc. Moreover, as previously mentioned, these new organisations would have to operate with different types of (often overlapping) IPRs (not just copyright), and deal with a variety of types of use of the IP-protected innovation. They would also have to work with a wide variety of industries, whereas most existing right management organisations tend to focus on one specific branch at a time, such as the music industry. All this might make it difficult to agree on royalties – especially as these would be based on FRAND principles, rather than on market influence and demand for innovation as is the case with the models used by most existing rights management organisations. In some cases, national legislation might also need to be adapted to integrate this model. Therefore, designing and testing the system proactively and well before any pandemic begins is of the highest importance, as multiple trials and reiterations might be needed in order to achieve a satisfactory result.

It might be difficult to arrange this type of licencing agreement on a compulsory basis – such agreements could be caught by the legal provisions for compulsory licensing (with all the administrative burdens that those entail). On the other hand, as we know, a voluntary tool poses the challenge that key players in the market must commit to it. Public pressure and brand values could be powerful tools in persuading organisations to participate. From a company or corporate law perspective, a requirement might also be contemplated at the level of corporate social responsibility, with companies being strongly recommended to participate in such efforts.

This type of semi-automatic mechanism for obtaining the permissions required for using certain innovations central to tackling a health emergency would be highly beneficial for the 3DP community, as it would make it easier to determine from whom to obtain licences and would also ensure that licences were affordable. Yet licensing IPRs – especially patents – and technologies might not be enough. As we have seen with the current ongoing pandemic, it is not enough, for instance, to free vaccines from patents to enable developing countries to reproduce the innovations involved. There is also a need to share valuable relevant trade secrets and know-know, as well as to develop tools to foster the building of capacity. As for the latter, digital manufacturing technologies like 3DP actually have considerable potential because of their flexibility. They do not require part-specific tooling, data can be sent through the internet, and different parts can be made in the same production run. This gives us even more reason to frame the legal system in such a way as to promote the development and use of these technologies. However, the fact that sharing IPRs is just not enough very much holds true also for 3DP, as IP does not provide 3D files, a print constellation or an actual “recipe” for how products should be made.

Overall, this proposed solution could enable companies and organisations to receive fair and reasonable royalties. Moreover, as people could be treated and protected, it could shorten the duration of lockdowns, considerably reducing both economic losses and the diminution of well-being. It would also ensure that companies were able to fully rely on IPRs during emergencies, thus securing revenues. In addition, since the medical industry is rather traditional and the majority of market share is held by large corporations, it could allow smaller, innovative and agile companies to enter the market and resolve some of the market needs caused by pandemics.

Another option for permitting commercial-scale use of patented technology without consent is government or Crown use, which is, for instance, provided for within UK patent legislation. This feature was developed initially by case-law relating to the Crown’s right to use an invention without the consent of the patentee and without paying compensation.^73^ The Patents, Designs and Trade Marks Act 1883, which is the basis of the modern provision, made Crown use part of statutory law, though compensation to the patentee then needed to be paid.^74^ Currently, Crown use of patented inventions is regulated by Secs. 55−59 of the UK Patents Act 1977 and permits “any government department and any person authorised in writing by a government department” to commit any of the acts listed in Sec. 55(1), namely making and using a patented invention without the consent of the right holder. Section 59 extends these powers even “[d]uring any period of emergency” where an emergency has been declared. The necessary authorisation in writing that third parties require in order to be able to commit any of the acts of use specified in Sec. 55(1) may be arranged before or after the patent is granted and may also be provided before or after the authorised acts have been committed.^75^ While Crown use specifically covers “the production or supply of specified drugs and medicines”, the general term “for the services of the Crown” is not exhaustively defined^76^ and may thus cover, for instance, PPE.^77^

The Court of Appeal has recently ruled that the authorisation given by government must not be general in its wording but rather “must be an authorisation to do acts in relation to a patented invention, not merely an authorisation to do acts”.^78^ Thus, the CIPA commentary notes that the decision may require contractors to

be advised to identify any need to use such inventions in advance wherever possible, and to secure an express authorisation from the relevant government department where necessary, noting that such departments are likely to encourage their contractors to pursue commercial licensing solutions in preference to the exercise of Crown user rights wherever possible.^79^

Liddicoat and Parish, however, suggest that this requirement would be rather straightforward to fulfil in the scenario they investigated, which was the COVID-19 pandemic.^80^ However, one element that might deter Crown use is that the royalty is negotiated after use; this may lead to hesitancy in employing this tool.^81^ To conclude, in assessing the viability of such provisions for this study, it is quite telling that the United Kingdom has not used this tool during the COVID-19 crisis.

## Conclusion

At the time of writing this article (summer 2022), there seems to be no full agreement yet on the proposed WTO vaccine patent waiver compromise.^82^ At the same time it is clear that the debated COVID IP waiver alone will not be a “silver bullet” that will automatically restore balance between the protection necessary to incentivise innovation on the one hand and fair global access on the other. But it is our hope that the current focus on the role of IP will trigger more nuanced debates that will ultimately enable us to improve pandemic preparedness, as well as to develop more innovative and sustainable pandemic responses for many sectors, including 3DP.^83^

More broadly, our analysis might very well find applications also outside the 3DP and medical pandemic context, as the issues raised are rather fundamental to the oft-discussed critical question of how to achieve a balance between IPR protection and access. As a provocative example, one could ask: What if the next pandemic or crisis were local, e.g. affecting only developed countries like the EU or the USA, instead of global? If we were to assume also that this local Western crisis led to a shortfall in critical innovations, which for some reason were mostly owned outside the EU/USA, such as by China, would Chinese companies freely allow the EU/USA to reproduce and use their protected innovations to deal with the crisis? These thoughts are not so futuristic after all. The supply of many raw materials is insecure in Europe, as clearly demonstrated also by recent developments affecting the political stability of Ukraine and Russia. Not only are over 80% of critical raw materials located outside the EU and the USA, but also most technologies to recycle and recover them are actually being developed (and protected) by non-European/American players. All of this does indeed force us to reconsider the magnitude of the problem, with the importance of further developments in this area becoming ever more palpable.^84^

More generally, every crisis, such as the COVID-19 pandemic, breeds opportunity. 3D printing technologies have demonstrated some of their incredible potential in an emergency. This article has sought to identify means of unleashing the potential benefits of this technology while ensuring legal certainty for users and fair consideration of property rights for right holders. Thus, cautious legislative changes are warranted and have been suggested here and elsewhere. However, the COVID crisis might also showcase the potential for right holders to consider 3DP and related technologies as a new form of distribution. The breakdown of traditional manufacturing and supply chains during the COVID-19 pandemic has shown how fragile these traditional forms of trade can be.^85^ In contrast, digital trading platforms could at least supplement the way right holders commercialise their products and provide an additional income stream. This, however, raises other legal questions (i.e. the legal nature of CAD files), regulatory issues (i.e. product liability) and commercial matters, which fall outside the scope of this paper. Approaches to tackling this and any future crisis may herald a new era of more sustainable digital distribution and trade.

## Footnotes

1 Chekurov and Salmi (2017), pp. 23–30.

2 Chekurov et al. (2021) Journal of Manufacturing Technology Management

3 Verboeket et al. (2021), pp. 25818–25834.

4 Gander (2015).

5 Valdes (2020) Journal of Business & Intellectual Property Law.

6 Mahr and Dickel (2020) Journal of Intellectual Property Law & Practice. The CEO of the company stated that a patent infringement case was never contemplated – https://www.theverge.com/2020/3/17/21184308/coronavirus-italy-medical-3d-print-valves-treatments.

7 Mäkitie et al. (2010).

8 Salmi (2021), p. 191.

9 Bhaskar et al. (2020).

10 Von der Leyen (2020).

11 Novak and Loy (2020).

12 Ford et al. (2020), pp. 1–7.

13 NESSIE (NA).

14 Beer et al. (2021).

15 GlobalData Healthcare (2021).

16 RSNA (2021).

17 OSMS 2022.

18 Salmi (2020), p. 4004.

19 Because of this rapid development and the crisis situation some countries have used accelerated and emergency approval processes for producing medical products via 3D printing (*see e.g.* Manero et al. (2020), p. 4634). In some countries, hospitals have used non-approved products, since these were better than no products at all (*see* Erikainen and Stewart (2020), p. 91). However, in most countries, the same approvals and protocols for 3D printing as in “normal” times have had to be followed (*see* Cooley LLP (2020).

20 Gordon 2020, p. 1613.

21 Ricketson (2015), paras 10.40 ff.

22 The Agreement mentions that Art. 31 relates to “Other Use Without Authorization of the Right Holder”, which is understood to refer to compulsory licensing. This is even more clearly the case with the introduction of Art. 31^bis^, which specifically mentions compulsory licensing.

23 The Agreement, however, specifically forbids the compulsory licensing of trade marks (Art. 21).

24 TRIPS (1994), Art. 1(1).

25 WTO (2017).

26 Houldsworth (2020).

27 Klopschinski (2020).

28 Apotex, the company in Canada producing generic drugs under this particular procedure, found it “to be highly inconvenient and commercially unviable” – Dutfield and Suthersanen (2020), p. 410.

29 Gurgula and Hull, p. 1248. *See also* Sinha 2021. Forthcoming, Cohen et al. (2022).

30 Firth (2009), p. 170. *See* also Gervais (2012), para 2.345; European Commission (2016), p. 144.

31 ‘t Hoen (2017), p. 7.

32 McMahon (2020), p. 146.

33 Van Zimmeren et al. (2022).

34 TRIPS, Art. 31(b) This requirement may be waived “in the case of a national emergency or other circumstances of extreme urgency or in cases of public non-commercial use”.

35 WIPO (2012).

36 WHO news (2021).

37 NIH COVID-19 Response 2020; Another example of an IP pool developed in the COVID-19 context is the “C_TAP”, which works through its implementing partners, namely the Medicines Patent Pool, the Open COVID Pledge, and the UN Technology Bank and Unitaid, to facilitate timely, equitable and affordable access to COVID-19 health technologies. WHO (2020).

38 NIH Essential.

39 *Ibid*.

40 For a general overview of IP pledges *see* Contreras and Jacob eds., (2017); Ehrnsperger and Tietze (2019).

41 Contreras et al. (2019). *See* *also* Contreras 2020, pp. 1146–1149.

42 Ford et al. (2020).

43 Open COVID Pledge.

44 For instance, IBM and Microsoft have pledged to license their entire patent portfolios. Intel has described the IP it has pledged as its “entire” IP portfolio (subject to a key limitation described in further detail below), which would include software. At the same time, however, the Open COVID Pledge is only for patents and copyright: trade secrets and trade marks are excluded. Thus, statements like Intel’s claim that its “entire” IP portfolio is included in the pledge must be read with this key limitation in mind. *See also* Kong and Warpula (2020).

45 Eduardo and Ramani (2020), pp. 367–384.

46 This is equally relevant for compulsory licensing, where the relevant national procedures for obtaining compulsory licences need to be adhered to – *supra* 3.1.

47 Lawder and Shalal (2022).

48 Draft Ministerial Decision on the TRIPS Agreement, Ministerial Conference Twelfth Session, Geneva, 12–15 June 2022, WT/MIN(22)/W/15/Rev.1, 17 June 2022.

49 Waiver from certain provisions of the TRIPS Agreement for the prevention, containment and treatment of COVID-19, Communication from India and South Africa, IP/C/W/669, 2 October 2020. Available at: https://docs.wto.org/dol2fe/Pages/SS/directdoc.aspx?filename=q:/IP/C/W669.pdf&Open=True.

50 *E.g.* Art. 53(c) EPC 2000.

51 *E.g.* Art. 52(2) and (3) EPC 2000.

52 Other examples that can be mentioned here are the functionality exclusions in EU trade mark and design law, Art. 7(1)(e)(ii) of Regulation (EU) 2017/1001 of the European Parliament and of the Council of 14 June 2017 on the European Union trade mark, OJ L 154, 16.6.2017, pp. 1–99; Art. 8(1) of Council Regulation (EC) No 6/2002 of 12 December 2001 on Community designs. OJ L 3, 5.1.2002 pp. 1–24.

53 Bently et al. (2010), p. 64.

54 In relation to patents *see* Machlup (1958), p. 21.

55 This, of course, depends on the complexity of the object to be 3D printed and how easy it would be for those seeking to 3D print it to reverse engineer it.

56 Bently (2011), pp. 331–332.

57 Bently and Sherman (2015), p. 326.

58 A two-step test for trademarks, *see* Art. 17 TRIPS.

59 Art. 13 TRIPS for copyright; Art. 17 TRIPS for trade marks, Art. 26 (2) for designs and Art. 30 TRIPS for patents. The terminology of these provisions is largely the same but with important differences that could lead to diverging interpretations.

60 Kur (2008), p. 9.

61 This text is modelled upon the exception provisions found in the Agreement on a Unified Patent Court, Art. 27.

62 WTO (2020a, b) WT/DS114/R.

63 *Ibid*, p. 155, 7.30.

64 *Ibid*, p. 155, 7.30.

65 *Ibid*, p. 152, 7.20. *See also* WTO (2020a, b) WT/DS160/R, p. 30, 6.97.

66 Correa (2020), p. 299.

67 Dinwoodie and Dreyfuss (2012), p. 62; Kur (2008), p. 24.

68 Correa points out that future panels are not bound by this interpretation and suggest that exceptions may be deemed limited based on the acts involved, the purpose and outcome of use of the invention, the persons invoking the exception or its duration – Correa (2020), p. 299.

69 Mendis et al. (2020).

70 Technical solutions for this purpose are already available on the market. *See* e.g. https://secured3d.com/, which offers secured cloud storage and streaming of 3D designs with monitoring, managing, and tracking of IP, as design files are printed inside and outside the company.

71 Ballardini and Lee (2017).

72 Pila (2020), p. 533.

73 Yang (2015), p. 399; Johnson (2020), p. 594.

74 *IPCOM GmbH & Co Kg v. Vodafone Group Plc & ors* [2021] EWCA Civ 205[130].

75 Sec. 55(6) UK Patents Act 1977. The general compatibility of retrospective authorisation with the TRIPS Agreement beyond cases of “national emergency or other circumstances of extreme urgency” and “public non-commercial use” as provided for in Art.31(b) TRIPS was questioned by the Court of Appeal recently – *IPCOM GmbH & Co Kg v. Vodafone Group Plc & ors* [2021] EWCA Civ 205 [150].

76 Sec. 55(1)(a)(ii) UK Patents Act 1977; *see also IPCOM GmbH & Co Kg v. Vodafone Group Plc & Ors* [2020] EWHC 132 (Pat) [188].

77 Lawder and Shalal (2022), p. 256.

78 *IPCOM GmbH & Co Kg v. Vodafone Group Plc & ors* [2021] EWCA Civ 205 [150].

79 CIPA (2020), Supplement, Commentary on Section 55.

80 Liddicoat and Parish (2021), p. 255.

81 Walsh et al. (2021), p. 404.

82 Lawder and Shalal (2022).

83 *Cf*. on the preceding debates: Matthews and Minssen (2021a, b) (Debating IP waivers…); *see also* Matthews and Minssen (2021a, b) (US COVID IP waiver, etc.).

84 *See* e.g. Bhaskar et al. (2020).

85 *Ibid*.
